# A Comparative Study of Ni-Based Catalysts Prepared by Various Sol–Gel Routes

**DOI:** 10.3390/molecules29174172

**Published:** 2024-09-03

**Authors:** Atheer Al Khudhair, Karim Bouchmella, Radu Dorin Andrei, Vasile Hulea, Ahmad Mehdi

**Affiliations:** 1 Department of Chemistry, College of Science, University of Kerbala, Karbala 56001, Iraq; 2 Department of of Dentistry, Al-Zahrawi University College, Karbala 56001, Iraq; 3 ICGM, University Montpellier, CNRS, ENSCM, 34095 Montpellier, France; karim.bouchmella@umontpellier.fr (K.B.); vasile.hulea@umontpellier.fr (V.H.); 4 ICSI Energy Department, National Research and Development Institute for Cryogenic and Isotopic Technologies, 240050 Ramnicu Valcea, Romania

**Keywords:** hydrolytic sol–gel, non-hydrolytic sol–gel, silica–alumina mixed oxides, heterogeneous catalysts, ethylene oligomerization

## Abstract

The use of heterogeneous catalysts to increase the development of green chemistry is a rapidly growing area of research to save industry money. In this paper, mesoporous SiO_2_-Al_2_O_3_ mixed oxide supports with various Si/Al ratios were prepared using two different sol–gel routes: hydrolytic sol–gel (HSG) and non-hydrolytic sol–gel (NHSG). The HSG route was investigated in both acidic and basic media, while the NHSG was explored in the presence of ethanol and diisopropyl ether as oxygen donors. The resulting SiO_2_-Al_2_O_3_ mixed oxide supports were characterized using EDX, N_2_ physisorption, powder XRD, ^29^Si, ^27^Al MAS-NMR and NH_3_-TPD. The mesoporous SiO_2_-Al_2_O_3_ supports prepared by NHSG seemed to be more regularly distributed and also more acidic. Consequently, a simple one-step NHSG (ether and alcohol routes) was selected to prepare mesoporous and acidic SiO_2_-Al_2_O_3_-NiO mixed oxide catalysts, which were then evaluated in ethylene oligomerization. The samples prepared by the NHSG ether route showed better activity than those prepared by the NHSG alcohol route in the oligomerization of ethylene at 150 °C.

## 1. Introduction

Ethylene oligomerization is one of the most important processes used to produce higher olefins. It provides the raw materials for the production of plastics, lubricants, surfactants, alcohols, etc. In the literature, many studies deal with the use of homogeneous and heterogeneous catalysts in ethylene oligomerization [1]. In accordance with the principles of sustainable chemistry, the development of heterogeneous catalysts and processes has been investigated in order to overcome problems and improve existing processes [2]. Several solid catalysts have been explored over the years as metal–organic frameworks, covalent organic framework materials and supported metal on inorganic porous supports, often prepared by a multi-step process [3]. For example, mesoporous aluminosilicates that contain nickel have demonstrated promising catalytic activity and selectivity in olefin oligomerization [3,4]. Since these catalysts with nickel ions also contain acid sites, they are actually bifunctional catalysts. It is commonly recognized that the isolated nickel ions contribute to the ethylene oligomerization process that produces 1-olefins, while the isomerization of primary olefins is catalyzed by acid sites. The result of these reactions is a combination of C4-, C6- and C8-olefins [5]. Oxides and mixed oxides with interesting chemical characteristics (acidity, basicity or redox properties) are used in heterogeneous catalysis, such as in oil refining, environmental catalysis and fine chemicals synthesis [4]. A wide range of materials could be used as supports on which the active phase is stabilized and dispersed. Simple metal oxides, including silica, alumina, aluminum silicates and titanates, are the most common [6]. Starting from metal molecular precursors, oxo bridges between metal atoms are formed by hydrolysis, and polycondensation reactions via the hydrolytic sol–gel process (HSG), which has been very successful in this area [7,8]. HSG offers many adjustable synthesis variables, such as the nature and concentration of precursors, the quantity of water, temperature, solvent, catalysis, drying and aging conditions. One of the major disadvantages of HSG is that it is a multi-step process and is more restrictive; the molecular precursors could have different reaction rates, leading to possible inhomogeneity [9,10]. In the case of mixed oxide synthesis, the homogeneity is clearly correlated to the two–steps of the HSG process: the hydrolysis and polycondensation rates of the precursors. Thus, to control the homogeneity in multi-component systems, the control of the reactivity of each precursor is necessary [9,11]. To resolve this problem, several methods have been investigated [12]. In the last three decades, another sol–gel synthesis pathway has been developed. Its originality comes from the chemical reactions involved in this process. The oxo bridges are formed by the reactions of metal chlorides with an oxygen donor other than water (for example, alkoxides, ethers, alcohols, etc.). The “non-hydrolytic” sol–gel (NHSG) term was proposed by Vioux and coworkers [13]. Since then, this process has been investigated and developed to prepare materials with different morphologies and physicochemical properties for various applications [14,15]. A sol–gel method is considered non-hydrolytic or non-aqueous if the oxygen donor used is not water and if no water is formed during the synthesis; therefore, it uses completely different condensation reactions from those used in an aqueous method. Many NHSG routes have been described that involve various precursors with various oxygen donors [12,16]. NHSG routes, particularly those that use alkoxides or organic ethers as oxygen donors to condense chloride precursors (“alkoxide route”) or ethers (“ether route”), have long been known as simple and efficient methods to prepare homogenous mixed oxide xerogels with mesoporous textures [4]. In comparison to the alkoxide and the ether routes, the alcohol route has received much less attention for the preparation of oxides and mixed oxides [12]. It is formed by the action of alcohols on halides. Bourget et al. reported the synthesis of silica by the alcohol route [17] using a solvent-free non-hydrolytic condensation reaction between silicon tetrachloride and benzyl alcohol as the oxygen donor. The reactions involved in the alcohol route depend on the nature of the alcohol—either primary, secondary or tertiary alcohol. A recent study showed that the oxygen donor has an influence on the morphology, the texture, the hydrophilicity/hydrophobicity and the catalytic activity of the final material [18].

In this work, we prepared SiO_2_-Al_2_O_3_ supports using classical HSG (acidic and basic media) and NHSG routes (alcohol and ether routes). Because SiO_2_-Al_2_O_3_ supports prepared by NHSG presented more regularly distributed samples and more acidity, they were selected to prepare SiO_2_-Al_2_O_3_-NiO mixed oxide catalysts and test their catalytic activity in the ethylene oligomerization reaction.

## 2. Results and Discussion

### 2.1. Synthesis and Characterization of Mixed Oxides

**Scheme 1:**
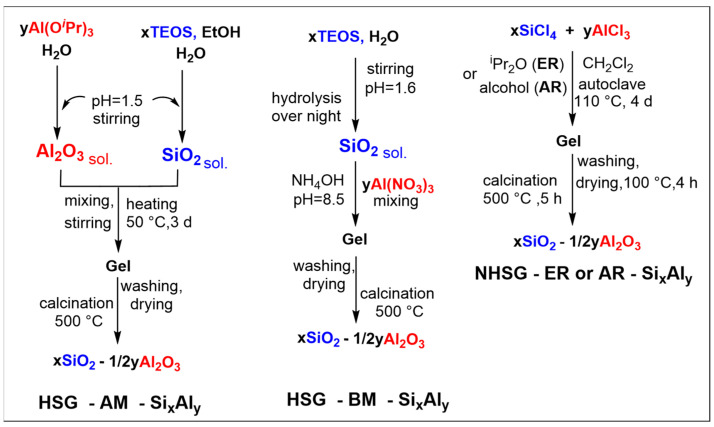
Experimental conditions for materials syntheses following the hydrolytic sol-gel process (HSG) in acidic Media (AM) and basic media (BM) and non-hydrolytic sol-gel using the ether route (ER) and alcohol route (AR) as oxygen donors.

Scheme 1 exhibited the experimental conditions for material syntheses following HSG in Acidic Media (AM) and basic media (BM), and NHSG using the ether route (ER) and alcohol route (AR) as oxygen donors.

The composition of samples was determined by EDX analysis. Texture and acidity were characterized for mixed oxides and catalysts, as shown in Table 1. The samples were labeled SixAly(Niz), where x, y and z represent the nominal SiO_2_, Al_2_O_3_ and NiO weight loadings, respectively.

**Figure 1:**
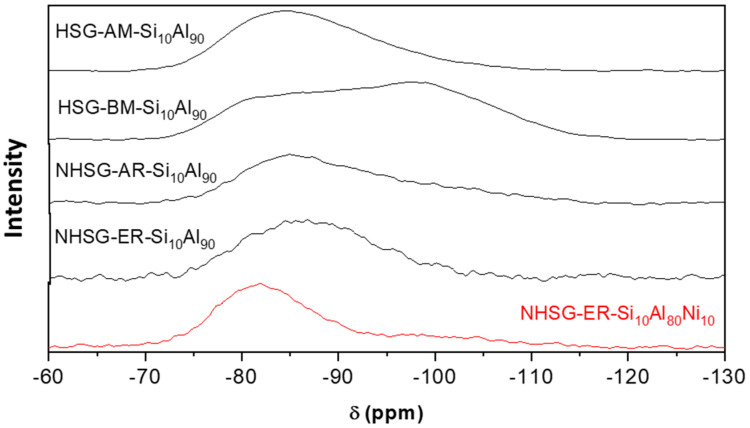
^29^Si CP−MAS NMR spectra of prepared Si_10_Al_90_ samples following the HSG in Acidic Media (AM) and basic media (BM) and NHSG using the ether route (ER) and alcohol route (AR) as oxygen donors.

The EDX demonstrates that the experimental weight percentages were close to the expected ones, based on the amounts of reactants, showing that all of the Si and Al atoms (for supports) and Si, Al and Ni atoms (for catalysts) were included in the final oxide. The same behavior was shown when silica-alumina and nickel were prepared by the sol-gel route [19]. The N_2_ adsorption–desorption measurements were performed, showing that the texture of all samples depended on the Si/Al ratio and the synthetic route. As shown in Table 1, the average pore diameter (Dp) ranged between 2.7 and 6.5 nm, indicating the mesoporosity of all samples. Samples prepared via HSG showed higher textural properties (in terms of S_BET_, Vp and Dp) than those obtained via NHSG. Agliullin et al. noticed the same observation when they used sol–gel processes to prepare porous silica–alumina [20]. For SixAlyNiz mixed oxides, samples prepared by NHSG (the ether route) showed better texture properties than those prepared by NHSG (the alcohol route). Alumina prepared by the ether route also showed a higher specific surface area than one prepared following the alcohol route [18]. The ^29^Si and ^27^Al solid-state NMR provides information on the structure of the aluminosilicate network. In the ^29^Si CP-MAS NMR spectra, all samples exhibited broad resonances (Figure 1), which are typical of amorphous materials [21,22]. 

In this work, the SixAly supports were composed of amorphous silica, and thus, only Q^N^ signals were expected. The signal is more or less wide (from −115 ppm to −70 ppm) depending on synthesis conditions for the aluminosilicate mixed oxides containing the same amount of Al (Si_10_Al_90_). The ^29^Si MAS NMR spectrum of the HSG-BM-Si10Al90 consisted of a broad, dissymmetric signal centered at about −95 ppm, indicating different environments of the Si atoms and suggesting a contribution of Q^2^ (−90 ppm), Q^3^ (−100 ppm) and possibly Q^4^ (−110 ppm) species.

**Figure 2:**
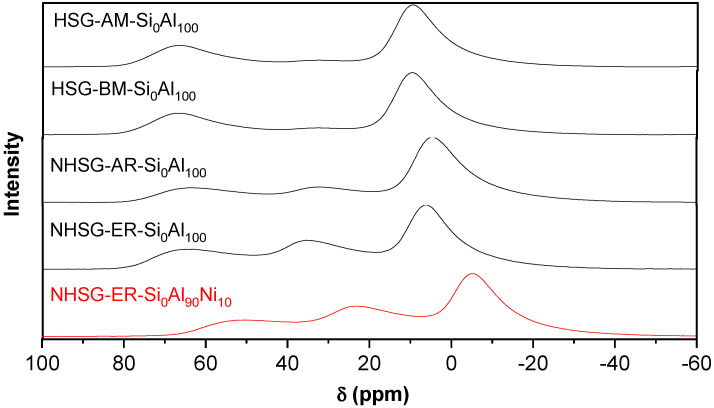
^27^Al MAS NMR spectra of prepared Si_0_Al_100_ samples following HSG in Acidic Media (AM) and basic media (BM) and NHSG using ether route (ER) and alcohol route (AR) as oxygen donors.

It must be noted that the CP-MAS sequence favors Si nuclei close to protons; thus, these spectra cannot be used to quantify the concentration of each site. Similar ^29^Si CP-MAS NMR spectra have been reported for mesoporous silica–alumina with a high Al content [23]. In amorphous silica–alumina, the incorporation of a large amount of aluminum in the silica network led to a significant low-field shift of the resonances, indicating the extensive formation of Si-O-Al bridges. A higher low-field shift was observed for the catalyst prepared by NHSG (in red, Figure 1), suggesting a better distribution of Al atoms in the silica matrix and a possible effect of Ni atoms in the electronic environment of Si atoms. The ^27^Al solid-state MAS NMR spectra (Figure 2) exhibited broad resonances around 70 ppm, 35 ppm and 5 ppm, ascribed to tetra-coordinated (Al^IV^), penta-coordinated (Al^V^) and hexa-coordinated (Al^VI^) aluminum sites respectively [24,25,26].

**Figure 3:**
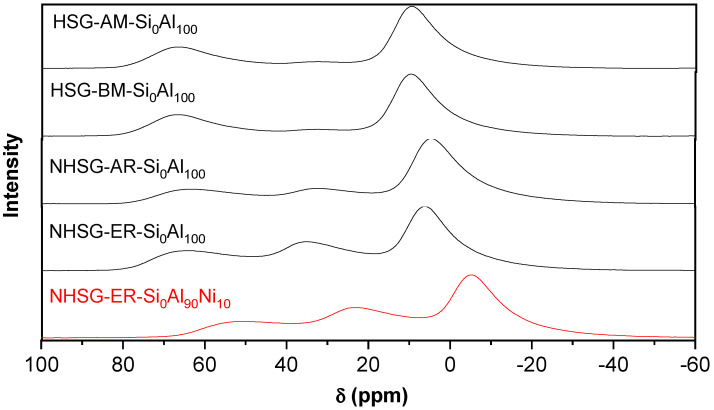
^27^Al MAS NMR spectra of prepared Si_10_Al_90_ samples following HSG in Acidic Media (AM) and basic media (BM) and NHSG using ether route (ER) and alcohol route (AR) as oxygen donors.

The tetra-coordinated and hexa-coordinated Al sites are the dominant Al coordinations on alumina and silica–alumina and are associated with introducing Brønsted and Lewis acidity on silica–alumina materials, respectively. The NHSG samples exhibited a higher peak intensity of penta-coordinated aluminum sites than those prepared by HSG. Rarely observed in silica–alumina, the Al^V^ sites are potential unsaturated Al species, like Al^IV^ ones, and thus, could be considered available for the formation of Brønsted acid sites [27]. This increase in Al^V^ concentration at the expense of Al^IV^ is promising for enhancing both surface Lewis acidity and Brønsted acidity in the NHSG samples. The signal corresponding to the Al^IV^ sites shifted upfield when the SiO_2_ loading increased (Figure 3). 

This effect has been previously ascribed to the decreased concentration of aluminum atoms around Al sites [23]. The addition of tetra-coordinated Si atoms in alumina leads to an increase in Al^IV^ and Al^V^ sites at the expense of Al^VI^ ones. This effect is more appreciable in the NHSG samples, suggesting the insertion of AlO_4_ tetrahedra in the silica network and a better homogeneity.

The X-ray diffractograms of the silica–alumina mixed oxides prepared by HSG and NHSG are shown in Figures S1 and S2. The XRD measurements showed that the structure of materials depended on the synthesis route. A gamma-alumina phase is detected in the alumina-rich samples prepared by the HSG routes (acidic and basic media) [28,29]. Samples prepared by the NHSG (ether and alcohol routes) are amorphous [30]. The diffractograms of the NHSG catalysts exhibited reflections at 2θ = 37.3°, 43.3°, 62.9° and 76.5°. These values are clearly related to the presence of NiO crystallites [31]. The estimation of particle sizes (from the width of the (200) reflection at 43.3°) is around 25 nm for these two alumina-rich catalysts (Figure S3). 

The acidity of supports (Si_x_Al_y_) and catalysts was determined using temperature-programmed desorption of ammonia (NH_3_-TPD). Unfortunately, this technique does not allow discrimination between the acidity that comes from Brønsted and Lewis acidic sites. Table 1 summarizes the total acidity measured for the prepared supports and catalysts. The supports (Si_x_Al_y_) prepared by NHSG showed higher acidity in terms of the total amount of desorbed NH_3_ and density of acidic sites than those prepared by the HSG routes. As shown by the ^27^Al solid-state NMR study, increasing the concentration of Al^IV^ and Al^V^ sites in the NHSG supports enhances their acidity. The SixAlyNiz ternary mixed oxides were prepared following the promising NHSG process. This good choice was confirmed by the high acidity values reached for the four catalysts. The samples prepared by the ether route showed slightly higher acidity than those prepared by the alcohol route. This phenomenon of increasing acidity with increasing Al_2_O_3_ content is characteristic of silica–alumina, independently of the synthesis process. It was employed for rhenium–silicon–aluminum mixed oxides prepared by a one-step NHSG [32]. Rajagopal et al. carried out a study on the acidity of silica–alumina supports with different percentages of Al_2_O_3_ prepared by HSG [33]. They demonstrated that with 25% alumina, the acidity was maximum, and the majority of Lewis acid sites were acidic. According to Scokart et al., the acidity of silica–alumina is greater for compositions containing 5 to 25% Al_2_O_3_ [34]. The high Brønsted acidity of silica–alumina is attributed to hydroxyl groups in the “Si-O-Al mixed phase”. The debate still exists on the structure of these sites in amorphous materials [35]. The Si-O-Al oxo bridge, thus formed by an intimate mixture of silicon and aluminum atoms, is charged more negatively than the oxygen of an Al-OH or a Si-OH. These species are, therefore, likely to generate stronger acidity. These results showed that the acidic properties of the NHSG samples are clearly linked to the structure of the amorphous silica–alumina and especially to the coordination of aluminum, as previously reported [36].

### 2.2. Catalyst Tests of Ternary Mixed Oxides

NHSG was used to prepare the ternary mixed oxides using a one-step process. This choice was due to the fact that this easy-to-implement process made it possible to prepare homogeneous mixed oxides with good texture and high acidity. Two compositions (Si/Al = 0 and 0.1) and two synthesis conditions were investigated (ether and alcohol routes), leading to the preparation of four catalysts (Table 2).

**Scheme 2:**
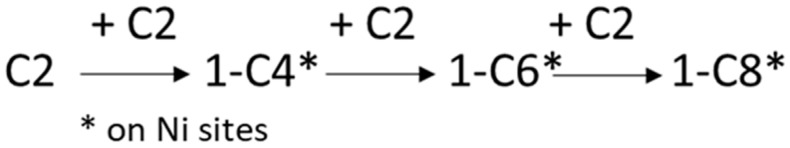
Reaction equation of the formation of C4, C6 and C8 olefins.

The Ni-based catalysts were tested in the ethylene oligomerization (Scheme 2).

The reaction was conducted at various experimental conditions: T = 150–350 °C, P = 3.5 MPa and mass hourly space velocity (WHSV) = 10 h^−1^ (Table S1). Under these conditions, butenes (C4), hexenes (C6) and octenes (C8) were the major products, and only small amounts of olefins with ≥10 carbon atoms were formed. In the C4 fraction, two n-butene isomers were detected: 1-C4 and 2-C4 (cis and trans stereoisomers). As shown in Table 2, the catalysts prepared by the ether route showed higher activity and conversion than those prepared by the alcohol route. However, the C4 selectivity was better for the SixAlyNiz catalysts prepared by the alcohol route.

As shown by XRD, all samples exhibited the presence of NiO crystallites. The higher activity of Si_0_Al_90_Ni_10_ and Si_10_Al_80_Ni_10_ catalysts prepared by the ether route cannot be explained by a texture effect as these two samples have a near-specific surface area compared to the samples prepared by the alcohol route. The TPD measurements showed that samples prepared by the alcohol route exhibited slightly less acidity and probably higher Brønsted acid sites. The lower catalytic activity of the samples prepared by the alcohol route could be attributed to the existence of isolated Ni^2+^ and Brønsted acidic sites not engaged in the formation of Ni^2+^-Brønsted catalytic sites [37]. We attributed the lower selectivity of the catalysts prepared by the ether route to their higher acidity. Indeed, it is well known that the formation of heavy oligomers from the C4 first product is due to strong acidity [38].

## 3. Materials and Methods

The SiO_2_-Al_2_O_3_ mixed oxides with two Si/Al ratios (0.0 and 0.1) were synthesized using the following as molecular precursors: tetraethylorthosilicate TEOS (Si(OC_2_H_5_)_4_ 98% from Sigma Aldrich, St. Louis, MO, USA), silicon tetrachloride (SiCl_4_ 99.98% from Alfa Aesar, Haverhill, MA, USA), aluminum trichloride (AlCl_3_ 99.98% from Alfa Aesar, Haverhill, MA, USA) and aluminum isopropoxide (Al(OCH(CH_3_)_2_)_3_ 98% from Sigma Aldrich, St. Louis, MO, USA). For the NHSG synthesis, the oxygen donors were dry ^i^Pr_2_O (H_2_O < 4 ppm) and dry CH_2_Cl_2_ (H_2_O < 6 ppm). They were purchased from Aldrich with 99% purity. The drying of these products was realized using an inert SPS (solvent purification system) from Innovative Technology.

### 3.1. Supports Synthesis

**HSG synthesis.** A colloidal SiO_2_ sol was prepared using TEOS, ethanol and water (1:1:2 volume ratio) in an acidic medium (pH = 1.5, adjusted with HCl). A transparent sol was obtained after stirring the solution for 1 h at room temperature. An Al_2_O_3_ sol was prepared following Yoldas’ method, starting from an Al(OCH(CH_3_)_2_)_3_ molecular precursor [28,39]. By mixing and stirring the SiO_2_ and Al_2_O_3_ sols, the SiO_2_-Al_2_O_3_ sol was prepared. After aging, the obtained gel was washed with solvents (ethanol, acetone and diethyl ether) successively. The material was dried at room temperature under vacuum for 1 h and for 4 h at 120 °C. The xerogel was crushed and calcined in air at 500 °C for 5 h (at 10 °C min^−1^).

**NHSG synthesis.** SiO_2_-Al_2_O_3_ mixed oxides were synthesized under an argon atmosphere (glove box and Schlenk line), starting from SiCl_4_ and AlCl_3_ as molecular precursors. The reactants were dissolved in 10 mL of dry CH_2_Cl_2_ inside an autoclave. Following the ether route, the number of moles of ^i^Pr_2_O was determined from the number of Cl and acac groups. However, in the alcohol route, an excess of alcohol (25 mL) was added. The reaction was realized in an autoclave at 110 °C for a duration of 4 days. The obtained materials were washed (CH_2_Cl_2_), dried under vacuum and then for 4 h at 120 °C, crushed and calcined in air at 500 °C for 5 h.

**Catalysts synthesis.** The SiO_2_-Al_2_O_3_-NiO mixed oxides with two Si/Al ratios (0.0 and 0.1) and a NiO content of 10 wt.% were synthesized using the NHSG process. The nickel bis(acetylacetonate) (Ni(acac)_2_, Alfa Aesar, Haverhill, MA, USA, 95%) was used as a molecular precursor and mixed with Si and Al ones at the beginning of the synthesis. All the rest of the procedure was the same as described for the NHSG SiAl supports.

### 3.2. Characterizations

The atomic percentages of Si and Al were obtained by energy-dispersive X-ray spectroscopy (EDX). Measurements were performed using an X-Max Silicon Drift detector mounted on an FEI Quanta FEG 200 scanning electron microscope. The data for each sample are the average of three different measurements to ensure accurate results in the analyses. X-ray powder diffractograms (XRD) were obtained on a Philips X-pert Pro II diffractometer using Kα copper Cu radiation (λ = 1.5418 Å) as a radiation source. The solid-state ^29^Si CP-MAS NMR spectra were recorded on a 59.6 MHz VARIAN VNMRS 300 spectrometer with a MAS (magic angle spinning) probe and 7.5 mm zirconia rotors, using a cross-polarization sequence (CP) with a 3 ms contact time, a 5 s recycling delay and a 5 kHz spinning frequency. The solid-state ^27^Al NMR spectra were recorded on a 104.26 MHz VARIAN VNMRS 400 spectrometer with a MAS (magic angle rotation) probe with 3.2 mm zirconia rotors, using a single pulse sequence with ^1^H decoupling, pulses of 1 μs (corresponding to a pulse angle of π/12), a recycling time of 1 s and a spinning frequency of 20 kHz. The N_2_ sorption experiments were carried out at 77 K on a Tristar instrument from Micrometrics. The outgassing of the calcined samples took place overnight at 150 °C under vacuum (2 Pa). The acidity was measured by temperature-programmed desorption of ammonia (NH_3_-TPD) on a Micromeritics AutoChem 2910 apparatus with a thermal conductivity detector.

## 4. Conclusions

Different sol–gel methods, in hydrolytic conditions (acid and basic media) and non-hydrolytic ones (EtOH and ether routes), were successfully used to easily prepare binary and ternary mixed oxides from non-expensive precursors. The silica–alumina supports with two silicon–aluminum ratios (0 and 0.1) were synthesized following these two processes. The sol–gel methods led to very good control of composition. The N_2_-adsorption–desorption showed that all samples were mesoporous. The texture strongly depended on the composition and on experimental synthesis (medium, solvent, molecular precursors, etc.). The XRD measurements showed that the structure of materials depended on the synthesis route. A gamma–alumina phase was detected only in the alumina-rich samples prepared by the HSG routes. The supports prepared by NHSG showed higher acidity in terms of total desorbed ammonia and density of acid sites than those prepared by HSG. A better distribution of Al atoms in the silica matrix and a higher acidity for the NHSG samples were revealed by solid-state NMR and NH_3_-TPD investigations, respectively. Considering these interesting and well-adapted properties for ethylene oligomerization and despite lower specific surface areas, the NHSG process was selected to prepare ternary SixAlyNiz mixed oxide catalysts. We showed that the catalysis activity in ethylene oligomerization was correlated with the synthesis route of sample preparation. Independent of the temperature reaction, the Ni-based catalysts prepared by the ether route showed higher activity than those prepared by the alcohol route, which exhibited higher selectivity in butene. We attributed these results to differences in the types of acidic sites correlated with Ni-active sites. NHSG has emerged as a simple but powerful method to prepare mesoporous multinary oxide materials, affording one-step syntheses of improved catalysts. In addition, its versatility allows the easy incorporation of dopants such as molybdenum oxide, providing unique opportunities for the discovery of new SiAlNiMo catalysts with superior catalytic performances in ethylene oligomerization.

## Figures and Tables

**Table 1:** Expected and experimental composition, texture and acidity of Si_x_Al_y_ samples prepared by different sol–gel routes and Si_x_Al_y_Ni_z_ samples prepared by NHSG (ether and alcohol routes).

Samples	Route	Expected and Experimental Compositions (wt.%)				Texture				Acidity	
		Si/Al	SiO_2_	Al_2_O_3_	NiO	SSA ^a^ (m^2^g^−1^)	Vp ^b^ (cm^3^g^−1^)	Dp ^c^ (nm)	SSA ^d^ µ/(m^2^g^−1^)	Total Desorbed-NH_3_ (mmol g^−1^)	Density (NH_3_ nm^−2^)
Si_0_Al_100_	Acidic HSG	0.0 (0.0)	0.0 (0.0)	100.0 (99.7)	-	375	0.4	4.8	40	1.0	1.6
Si_0_Al_100_	Basic HSG	0.0 (0.0)	0.0 (0.0)	100.0 (99.7)	-	340	0.4	4.0	42	0.9	1.6
Si_0_Al_100_	EtOH NHSG	0.0 (0.0)	0.0 (0.0)	100.0 (99.7)	-	360	0.2	3.1	55	2.8	4.7
Si_0_Al_100_	Ether NHSG	0.0 (0.0)	0.0 (0.0)	100.0 (99.9)	-	297	0.4	4.0	35	2.9	5.9
Si_10_Al_90_	Acidic HSG	0.1 (0.1)	10.0 (10.3)	90.0 (89.7)	-	286	0.3	4.4	70	1.3	2.7
Si_10_Al_90_	Basic HSG	0.1 (0.1)	10.0 (10.2)	90.0 (89.8)	-	400	0.4	3.6	65	1.4	2.1
Si_10_Al_90_	EtOH NHSG	0.1 (0.1)	10.0 (9.9)	90.0 (90.1)	-	271	0.4	5.8	60	2.8	6.2
Si_10_Al_90_	Ether NHSG	0.1 (0.1)	10.0 (9.8)	90.01 (90.2)	-	170	0.3	6.4	64	3.0	10.6
Si_0_Al_90_Ni_10_	Ether NHSG	0.0 (0.0)	0.0 (0.0)	92.7 (92.9)	7.3 (7.1)	348	0.4	6.5	59	4.8	11.6
Si_10_Al_80_Ni_10_	Ether NHSG	0.1 (0.1)	9.3 (6.3)	83.6 (86.5)	7.1 (7.2)	395	0.3	3.5	45	4.4	6.7
Si_0_Al_90_Ni_10_	EtOH NHSG	0.0 (0.0)	0.0 (0.0)	92.6 (92.8)	7.4 (7.2)	368	0.3	3.1	57	4.3	8.8
Si_10_Al_80_Ni_10_	EtOH NHSG	0.1 (0.1)	9.1 (6.5)	83.8 (86.2)	7.1 (7.3)	286	0.3	2.7	62	4.2	7.0

^a^ Specific surface area. It was determined by the BET method in the 0.05–0.25 P/P_0_ range. ^b^ Total pore volume at P/P_0_ = 0.98. ^c^ Average pore diameter 4 Vp/S_BET_. ^d^ From t-plot analysis. Total acidity was determined by NH_3_-TPD in the range of 100–900 °C.

**Table 2:** Catalysis activity in ethylene oligomerization of Ni-based catalysts.

Catalyst	Si/Al	Conv. (%)	Activity (mmol g^−1^ h^−1^)	Selectivity to (wt.%)		
				C4	C6	C8
NHSG-AR-Si_0_Al_90_Ni_10_	0.0	49	175	82	13	3
NHSG-AR-Si_10_Al_80_Ni_10_	0.1	44	157	78	15	4
NHSG-ER-Si_0_Al_90_Ni_10_	0.0	73	261	69	15	3
NHSG-ER-Si_10_Al_80_Ni_10_	0.1	79	282	72	20	5

Conditions: temp. = 150 °C, P = 3.5 MPa, reaction time = 65 min and WHSV =10 h^−1^.

## Data Availability

Not applicable.

## Supplementary Materials

The following supporting information can be downloaded at https://www.mdpi.com/article/10.3390/molecules29174172/s1, Figure S1: XRD patterns of SixAly supports; Figure S2: XRD patterns of SixAly supports; Figure S3: XRD patterns of SixAlyNiz catalysts prepared by NHSG. Table S1: Influence of reaction temperature on the catalytic performances of SixAlyNiz catalysts; Scheme S1: Simplified scheme of the main reactions involved in ethylene oligomerization.
