# Supplementary material for: A Comparative Study of Ni-Based Catalysts Prepared by Various Sol–Gel Routes
Source: Molecules. 2024 Sep 3;29(17):4172. doi: 10.3390/molecules29174172 (PMC11397447; doi:10.3390/molecules29174172)
Supplement: Supplementary file 1 [file molecules-29-04172-s001.zip › molecules-3092395-supplementary.pdf]

Electronic Supplementary Information

A comparative study of Ni-based catalysts prepared by various sol-gel routes

Atheer Al Khudhair*[1,2], Karim Bouchmella[3], Radu Dorin Andei[4], Vasile Hulea[3] and Ahmad Mehdi*[3]

[1] Department of Chemistry, College of Science, University of Kerbala, Karbala 56001, Iraq
[2] Department of of Dentistry, Al-Zahrawi University College, Karbala 56001, Iraq
[3] ICGM, University Montpellier, CNRS, ENSCM, 34095 Montpellier, France; karim.bouchmella@umontpellier.fr (K.B.); vasile.hulea@umontpellier.fr (V.H.)
[4] ICSI Energy Department, National Research and Development Institute for Cryogenic and Isotopic Technologies, 240050 Ramnicu Valcea, Romania; radu.dorin@icsi.ro

* Correspondence: atheer.h@uokerbala.edu.iq (A.A.K.); ahmad.mehdi@umontpellier.fr (A.M.)

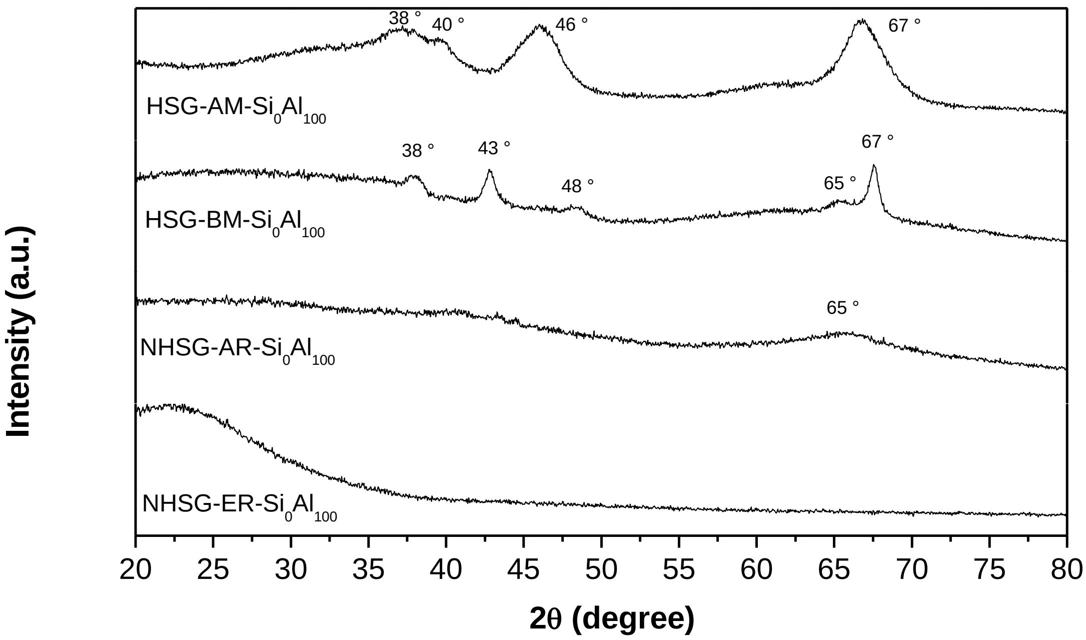

Figure S1: XRD patterns of SixAly supports.

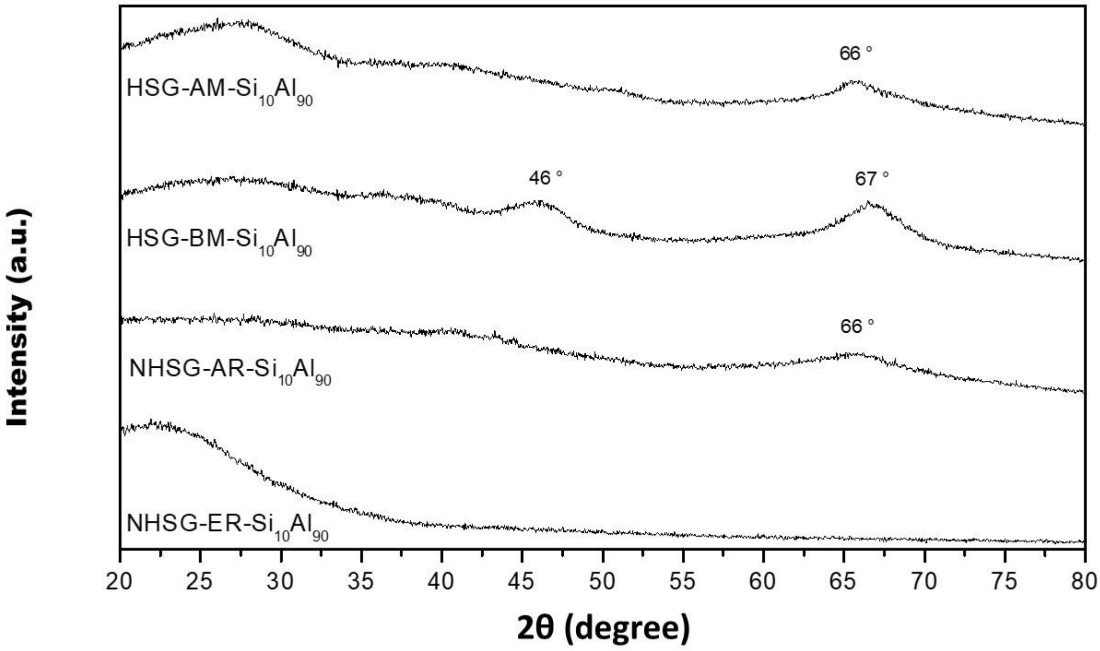

Figure S2: XRD patterns of SixAly supports.

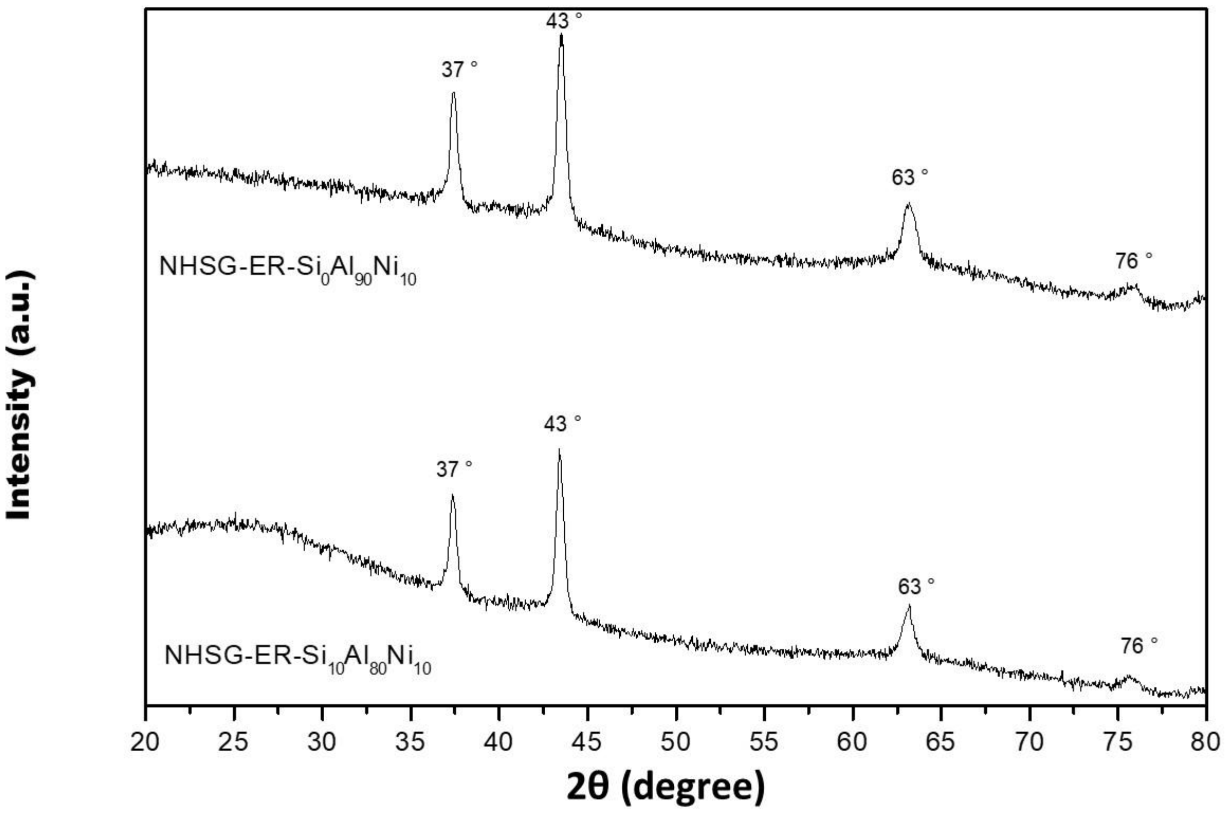

Figure S3: XRD patterns of $Si_xAl_yNi_z$ catalysts prepared by NHSG.

Table S1: Influence of reaction temperature on the catalytic performances of SixAlyNiz catalysts.[a]

Catalyst	T ($^\circ$C)	C2 conversion (%)	Product distribution (wt%)				Activity[c] (mmol g^{-1} h^{-1})	Route
			C4	C6	C8	Other [b]		
	150	48	82	13	3	1	170	
	200	53	85	14	4	3	182	
$Si_0Al_{90}Ni_{10}$	250	58	87	16	6	4	184	EtOH
	300	60	84	17	8	6	189	
	350	69	30	04	5	55	191	
	150	45	77	14	6	2	156	
	200	49	79	16	5	3	159	
$Si_{10}Al_{80}Ni_{10}$	250	53	82	18	7	4	163	
	300	58	80	13	8	5	168	EtOH
	350	67	26	6	3	58	171	
	150	76	70	18	08	03	271	
	200	88	66	22	08	04	314	
$Si_0Al_{90}Ni_{10}$	250	92	63	24	09	04	329	$^{i}Pr_2O$
	300	95	62	23	09	07	339	
	350	96	27	05	01	67	343	
	150	77	69	11	06	15	275	
	200	78	65	12	06	17	279	
$Si_0Al_{90}Ni_{10}$	250	80	61	15	07	19	286	$^{i}Pr_2O$
	300	85	34	20	06	18	304	
	350	92	27	03	01	69	329	

a Reaction conditions: P = 3 MPa, WHSV = 10 h− 1, reaction time = 1 h.
b Alkanes and uneven olefins.
c Average specific activity (mmol of C_2H_4 converted per gram of catalyst per hour).

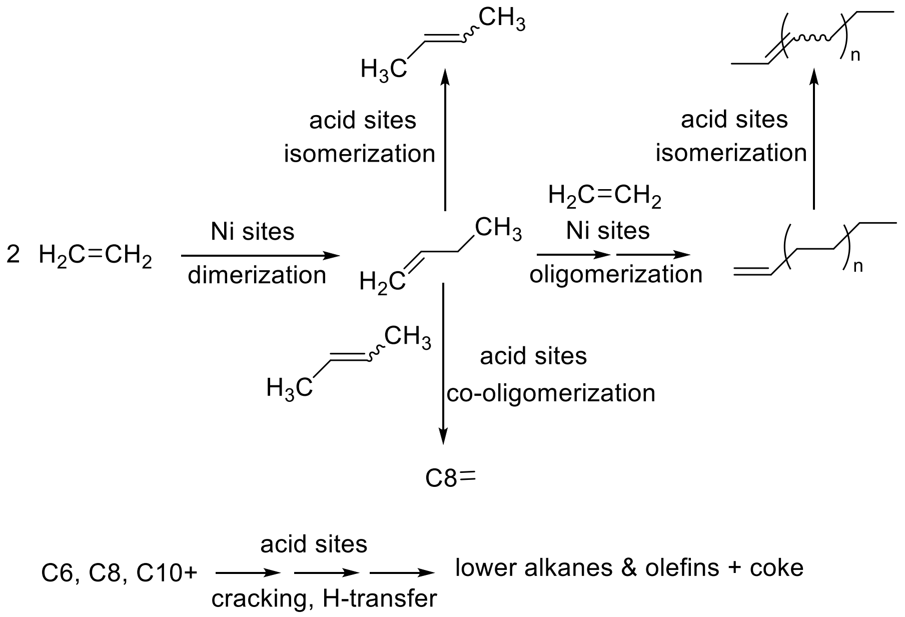

Scheme S1: Simplified scheme of the main reactions involved in ethylene oligomerization.
